# Guillain-Barré syndrome following snake bite: An unusual complication

**DOI:** 10.4103/0972-2327.61284

**Published:** 2010

**Authors:** Abhishek Srivastava, A. B. Taly, Anupam Gupta, Aumir Moin, T. Murali

**Affiliations:** Department of Physical Medicine and Rehabilitation, Kokilaben Dhirubhai Ambani Hospital and Medical Research Institute, 400 053, Mumbai; 1 Departments of Neurology, National Institute of Mental Health and Neuro Sciences (NIMHANS), 560 029, Bangalore; 2 Psychiatric and Neurological Rehabilitation, National Institute of Mental Health and Neuro Sciences (NIMHANS), 560 029, Bangalore; 3 Department of Neurology, Apollo Hospital, Mysore, India

**Keywords:** Guillain-Barrésyndrome, neuropathy, snake bite, tetanus toxoid

## Abstract

A 40-year-old man presented with a nonhealing wound on the left ankle for the last 5 weeks, a tingling sensation in both hands for 20 days, and weakness in all four limbs for 10 days. He had been bitten by a snake while working in a sugarcane field 6 weeks earlier and had received tetanus toxoid and anti–snake venom on the day of the bite. He had clinical, biochemical, and electrophysiological features of Guillain-Barré syndrome, with motor and sensory neuropathy—primarily suggestive of demyelination with secondary axonal degeneration. Recognition of this unusual complication following snake bite or use of anti–snake venom / tetanus toxoid has considerable epidemiological, therapeutic, and prognostic significance.

## Introduction

Guillain-Barré syndrome (GBS) is preceded by various antecedent events in over 50% of individuals. GBS can occur after viral or bacterial infections, in autoimmune diseases, after administration of certain drugs and vaccines, following surgery or organ transplantation, or following snake bite.[1] Till date only one case has been reported following snake bite; this was from Chinese Taipei in 1996.[2] In this article, we present one such case of GBS following snake bite.

## Case Report

A 40-year-old man, a farmer by profession, presented with a nonhealing wound on the left ankle that had been present for the last 5 weeks, a tingling sensation in both hands and feet for the last 20 days, and weakness in all four limbs of 10 days’ duration. He had been bitten by a snake while working in a sugarcane field 6 weeks earlier. He had received one dose of tetanus toxoid and two doses of anti–snake venom at a local hospital on the day of the bite. After about 2 weeks, he experienced tingling and numbness distally in all four extremities, followed by the development of weakness in all four limbs over the next week. He was referred to our institute for a neurological opinion 6 weeks after the snake bite. There was no history of dysphagia, dysphonia, diplopia, or bowel and bladder involvement. The past history and family history were noncontributory. He had a nonhealing wound over the left ankle that was oval in shape and 4×3 cm in size, with necrotic slough at the margins. Cranial nerves examination was normal except for bilateral facial weakness. The patient had sensory impairment in both lower limbs below the knee, generalized hypotonia and areflexia, reduced hand grip strength, and grade 3–4/5 Medical Research Council (MRC) motor power in both lower limbs. The autonomic functions were normal. Routine hemogram and biochemistry were unremarkable. CSF revealed albumino-cytological dissociation (proteins: 117 mg/dl, cell count: 2 lymphocytes/mm^3^). Serum creatine kinase was 189 U/l. Sensory nerve conduction study showed absent sensory nerve action potential (SNAP) in the median, ulnar, and sural nerves. Motor conduction of median, ulnar, and common peroneal nerves showed reduced amplitude of evoked motor response (EMR), prolonged distal latencies, and reduced velocities. Repetitive nerve stimulation test did not show any decremental response. The features were suggestive of motor and sensory neuropathy—primarily demyelination with secondary axonal degeneration.

The patient was diagnosed to have GBS and was treated with plasmapheresis. He underwent a short course of inpatient rehabilitation. At the time of discharge the wound on the ankle had healed and he was independent in the activities of daily living and ambulation. At follow-up after 6 months, he had resumed working in the fields though he still had occasional tremors of his hands and pain in the calves. Repeat nerve conduction study revealed improvement in amplitude of EMR, distal latencies, and conduction velocities.

## Discussion

The features of GBS in our patient could be attributed to the snake bite or the administration of tetanus toxoid or anti–snake venom. To the best of our knowledge only one case of GBS following snake bite has been reported in the literature.[1] One case each has been reported after tetanus toxoid administration[3] and after administration of combined diphtheria and tetanus toxoid.[4] No case has been reported to date following administration of anti–snake venom.

The neuromuscular complications of snake bite include neuromuscular junction transmission abnormality, which can be caused by elapid and some sea snake venom, and myotoxicity, which can be caused by elapid as well as viper venom. Chuang *et al*.[1] described a patient who developed axonal GBS following the bite of a Formosan krait. The patient presented with symmetric paresis and sensory signs in the upper and lower limbs, autonomic dysfunction, facial nerve involvement, and mild elevation of cerebrospinal fluid (CSF) protein at about 4 weeks after the bite, but had good functional recovery. Electrodiagnostic studies revealed profound sensory and motor polyneuropathy. Repeat electrophysiologic examination confirmed nerve regeneration. One case of axonal sensory motor neuropathy following snake bite has been reported from India[5] against the background of a sepsis syndrome, with sparing of the cranial nerves but with autonomic dysfunction. CSF study, even at 2 weeks after the illness, was normal and favored critical illness neuropathy rather than GBS.[6]

Newton and Janati[3] reported a case of GBS that developed after the injection of pure tetanus toxoid. They demonstrated a hypersensitive lymphoblastic transformation occurring in response to purified tetanus antigen; also, typing for disease-associated antigens was homozygous for HLA-B8. The National Immunization Program, Centers for Disease Control and Prevention, USA,[7] had conducted a study based on previous active surveillance epidemiological studies of GBS and vaccination history and reported a background rate of 0.3 cases of GBS per million person-weeks.

## Conclusion

GBS can occur following a snake bite or after the administration of tetanus toxoid but, due to poor documentation and reporting, the actual incidence might be much more than the reports in the literature suggest. There have been cases where clinical, biochemical, and electrophysiological studies were all suggestive of GBS, without the history of any antecedent factor other than snake bite or administration of tetanus toxoid. In our patient also, considering the temporal association of the symptoms with the history of snake bite and the administration of anti–snake venom / tetanus toxoid, as well as the absence of any other antecedent event, we considered the GBS as being secondary to one of these factors.
